# Intraspecies Prion Transmission Results in Selection of Sheep Scrapie Strains

**DOI:** 10.1371/journal.pone.0015450

**Published:** 2010-11-16

**Authors:** Takashi Yokoyama, Kentaro Masujin, Mary Jo Schmerr, Yujing Shu, Hiroyuki Okada, Yoshifumi Iwamaru, Morikazu Imamura, Yuichi Matsuura, Yuichi Murayama, Shirou Mohri

**Affiliations:** Prion Disease Research Center, National Institute of Animal Health, Tsukuba, Japan; Ames Laboratory, Iowa State University, Ames, Iowa, United States of America; Creighton University, United States of America

## Abstract

**Background:**

Sheep scrapie is caused by multiple prion strains, which have been classified on the basis of their biological characteristics in inbred mice. The heterogeneity of natural scrapie prions in individual sheep and in sheep flocks has not been clearly defined.

**Methodology/Principal Findings:**

In this study, we intravenously injected 2 sheep (Suffolk and Corriedale) with material from a natural case of sheep scrapie (Suffolk breed). These 3 sheep had identical prion protein (PrP) genotypes. The protease-resistant core of PrP (PrPres) in the experimental Suffolk sheep was similar to that in the original Suffolk sheep. In contrast, PrPres in the Corriedale sheep differed from the original PrPres but resembled the unusual scrapie isolate, CH1641. This unusual PrPres was not detected in the original sheep. The PrPres distributions in the brain and peripheral tissues differed between the 2 breeds of challenged sheep. A transmission study in wild-type and TgBoPrP mice, which overexpressing bovine PrP, led to the selection of different prion strains. The pathological features of prion diseases are thought to depend on the dominantly propagated strain.

**Conclusions/Significance:**

Our results indicate that prion strain selection occurs after both inter- and intraspecies transmission. The unusual scrapie prion was a hidden or an unexpressed component in typical sheep scrapie.

## Introduction

Transmissible spongiform encephalopathies (TSEs) are fatal neurodegenerative disorders caused by prions [1] and include scrapie in sheep and goats, bovine spongiform encephalopathy (BSE) in cattle, and Creutzfeldt-Jakob disease (CJD) in humans. Although the etiopathogenesis of prion diseases has not been fully elucidated, it is considered that these diseases result from the formation of an abnormal variant of the cellular isoform of prion protein (PrP^C^); the abnormal isoform (PrP^Sc^) and PrP^C^ exhibit different secondary and tertiary structures, and undergo different post-translational conformational changes [2]. PrP^Sc^ is distinguished from PrP^C^ on the basis of protease resistance: protease partly degrades PrP^Sc^ to form a protease-resistant C-terminal core fragment (PrPres), that has an unglycosylated form with a molecular weight of 19–21 kDa. PrPres obtained from different prion isolates have different N-terminal ends [3], [4]. Furthermore, in atypical scrapie sheep, a different size PrPres fragment along with an another 10–12 kDa band is observed [5].

The identification of BSE and variant CJD has raised important food-safety issues [6]. The origin of BSE is obscure, but BSE-causing prions may have arisen from a sheep scrapie agent. Further, BSE may have been transmitted to small ruminants via contaminated feed stuffs. BSE affected goats have been occasionally reported [7]. Therefore, it is important to elucidate the biological and molecular basis of sheep scrapie strains in their natural hosts.

Scrapie prions are classified into many different strains on the basis of the incubation period, lesion profile, and PrP^Sc^ distribution in inbred mice [8]. Conformational differences in PrP^Sc^ structure may contribute to strain variations [9], [10], [11]. The heterogeneity of natural sheep scrapie is reflected in the results of PrP^Sc^ molecular profiling [12]. However, limited information is available regarding the pathogenesis of scrapie prion strains in their original host–sheep.

The unusual scrapie isolate CH1641 and the PrPres that causes BSE (l-type PrPres) have similar molecular weights [13], which are lower than the molecular weight of the PrPres (h-type) that causes typical scrapie [14], [15], [16], [17]. Furthermore, CH1641 prions have a protein fragment produced by the C-terminally cleavage of PrPres, designed as PrPres #2 (molecular weight, 14 kDa) [18]. A transmission study in ovine transgenic mice has revealed differences between CH1641 scrapie and BSE [18]. Recently, natural cases of CH1641-like scrapie, a rare disease, have been reported[14], [17]. Prion strain diversity and TSE pathogenesis should be studied in sheep, and the findings obtained may help reveal the origin of BSE.

In this study, we conducted a transmission study by injecting prions obtained from a natural case of typical sheep scrapie (G3571; a Suffolk sheep) into sheep of different breeds (Suffolk, #2314 and Corriedale, #294) but with identical PrP genotypes with regard to the open-reading frame. The sheep Suffolk #2314 will be referred as the “Suffolk sheep” not to be confused with the original [G3571] sheep. The PrPres that accumulated in the Suffolk sheep was similar to the PrPres of typical sheep scrapie. The PrPres that accumulated in the Corriedale sheep was similar to that of the unusual CH1641-like prion. After transmission of these scrapie agents to rodents, the complexity of these prions in their natural hosts was observed. Interspecies prion transmission has resulted in the selection or mutation of prion strains. In this study, we showed that unusual scrapie prions were present in a case of typical scrapie, and that intraspecies transmission of the prions from this sheep resulted in the selection of a strain of PrPres that was different from the original strain.

## Methods

### Ethics Statement

The study protocol was approved by the Animal Ethics Committee (approval ID: 153, 404 and 520) and Animal Care and Use Committee (approval ID: 04-III-6) of the National Institute of Animal Health.

### Sheep scrapie

A case of naturally occurring scrapie (G3571) was detected in Ohio, USA in 1998 and used in this study. This sheep showed ataxia and loss of fleece, and was diagnosed with scrapie on the basis of (a) neuronal vacuolation observed on histopathological examination and (b) the presence of PrPres in the brain and tonsils, as observed on Western blot (data not shown). Brain tissue from this sheep was homogenized in phosphate-buffered saline (PBS) and centrifuged at 3,000×*g* for 10 min. The supernatant was intravenously inoculated into 4 sheep (three of the Suffolk breed and the other of the Corriedale breed) obtained from a historically scrapie-negative flock in Japan. The experimentally challenged sheep were monitored daily, and samples were collected after the sheep showed clinical signs of scrapie. Unfortunately, the 2 of the Suffolk sheep died accidentally at 7 and 259 days post inoculation. One sheep (259 days post inoculation) harbored PrPres in the spleen and tonsil. However, these 2 Suffolk sheep were negative for PrPres in the brains, and were excluded from this study.

### PrP genotype of sheep

PrP genotyping of sheep was performed as described previously [19]. All the sheep used in this study were homozygous for polymorphisms of codons 112, 136, 154, and 171 in the PrP gene; these codons encode the amino acids Met, Ala, Arg, and Gln, respectively.

### Bioassay

We intracerebrally inoculated 4-week-old female wild-type ICR mice [20] and bovinized transgenic mice (TgBoPrP) [21] with 20 µl of 10% brain homogenates obtained from the scrapie-affected sheep. TgBoPrP mice that expressing bovine PrP are highly susceptible to natural sheep scrapie as well as bovine BSE [22]. Phenotypic analysis of TgBoPrP mice with scrapie isolates may help determine the origin of BSE. When the inoculated mice showed clinical signs of terminal disease, they were sacrificed under anesthesia, and their brains were collected and processed for PrPres detection and examination for pathology. The brains of mice that died of unknown causes were examined as well for the presence of PrPres by Western blot.

### Sample preparation for Western blot

PrPres-enriched samples of brain tissue were prepared using a previously described procedure [23]. In brief, the brain samples were homogenized in a detergent buffer containing 2% sulfobetaine 3–14 and 0.5% *N*-lauroyl sarcosinate (sarkosyl) and then incubated with 500 µg/ml collagenase, followed by incubation with 40 µg/ml proteinase K (PK) at 37°C for 30 min. PK digestion was terminated by the addition of 2 mM Pefablock (Roche), and the sample was precipitated by mixing it with a butanol-methanol (5∶1) mixture and then centrifuging it at 20,000×*g* for 10 min. The pellet was subjected to Western blot.

PrPres-enriched samples were prepared from peripheral tissues as described previously [19]. In brief, minced tissues (200 mg) were homogenized in 50 mM Tris-HCl (pH 7.5) containing 2% (v/v) Triton X-100, 0.5% (v/v) sarkosyl, 100 mM NaCl, 5 mM MgCl_2_, 20 mg collagenase, and 40 µg DNase I and then incubated at 37°C for 2 h. The homogenate was digested with 80 µg PK at 37°C for 1 h and then centrifuged at 68,000×*g* for 20 min at 20°C. The resulting pellet was suspended in 6.25% sarkosyl in 10 mM Tris-HCl (pH 7.5) and centrifuged at 9,000×*g* for 5 min. Sodium phosphotungstate was added to the supernatant to achieve a final concentration of 0.3%; the resultant solution was incubated at 37°C for 30 min with constant rotation. After incubation, the solution was centrifuged at 20,000×*g* and 20°C for 30 min, and the pellet obtained was subjected to western blotting.

### Western blot analysis

Western blot analysis was carried out as described previously with monoclonal antibodies (mabs) T2 [23], SAF-84 (SPI bio), and P4 (R-Biopharm AG). Mabs P4, T2 and SAF-84 recognized subregion of 89–104, 136–143, and 163–173 of sheep PrP, respectively. For PrPres glycoform analysis, the relative quantities of the 3 PrPres bands were measured using Fluorochem software (Alpha-Innotech) after background subtraction. For band-profile analysis, only samples within the linear range, i.e., those with unsaturated signal intensities were used. PrPres from mouse-adapted scrapie (Obihiro strain) [24], C-BSE (natural Japanese case), and H-type atypical BSE (courtesy of Dr. S. Czub, Canadian Food Inspection Agency) were used as controls.

### Deglycosylation of PrPres

The PK-digested brain samples were denatured in glycoprotein-denaturing buffer (0.5% SDS, 1% β-mercaptoethanol; New England Biolabs) at 100°C for 10 min prior to incubation with peptide N-glycosidase F (PNGase F; New England Biolabs) at 37°C for 2–4 h. The reaction was terminated by denaturation by boiling in SDS.

### Pathology and Immunohistochemistry

Brain samples were fixed in 10% buffered formalin, dehydrated, and embedded in paraffin wax for examination for pathology. The samples were then sectioned, and the sections were subjected to hematoxylin and eosin or immunohistochemical staining. For detection of immunolabeled PrP^Sc^, dewaxed sections were pretreated with chemical solutions as described previously [25]. The mabs SAF-84 (SPI-Bio) or T1 [26] were used for immunolabeling.

## Results

### Intraspecies transmission of sheep scrapie

The scrapie isolate of G3571 was transmitted intravenously to 2 sheep of different breeds, both of which developed ataxia, abnormal gait, and debilitation but no loss of fleece. The incubation period differed between the 2 sheep (incubation period: Suffolk, 847 d; Corriedale, 1,666 d) (Table 1). Both the donor and recipient sheep had an identical PrP genotype.

**Table 1:** Summary of intraspecies transmission of sheep scrapie.

Sheep no.	Breed	PrP genotype^1^	Scrapie challenge^2^	Incubation period	PrPres profile^3^	PrPres #2^4^
G3571	Suffolk	MARQ/MARQ	natural case	1 y 5	high-type PrPres	-
#2314	Suffolk	MARQ/MARQ	G3571, i.v.	847 d	high-type PrPres	-
#294	Corriedale	MARQ/MARQ	G3571, i.v.	1,666 d	low-type PrPres	+

1 PrP amino acid sequence at 112, 136, 154, and 171.

2 Brain homogenates from G3571 were inoculated intravenously into 2 sheep (#2314 and #294).

3 Classified on the basis of the molecular weight of unglycosylated PrPres.

4 Existence of 14-kDa fragment of PrPres.

5 Estimated ages of diseased sheep.

### Characterization of PrPres in sheep brain

**Figure 1:**
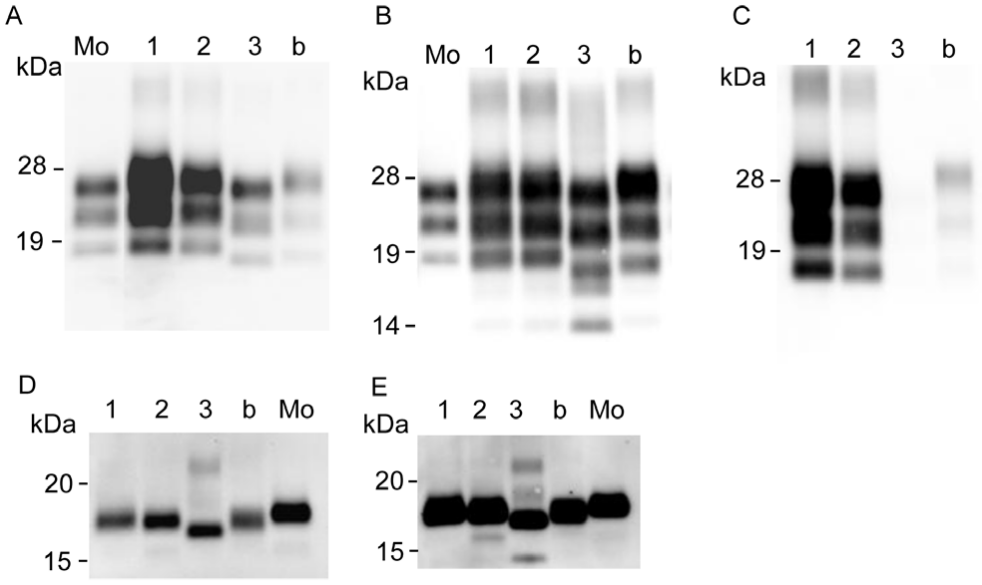
Western blotting analysis of PrPres in scrapie sheep brain. Obex homogenates were subjected to analysis by Western blot. Each lane contained 0.5 mg sheep brain equivalent sample. Lane 1: G3571, lane 2: #2314 (G3571-inoculated Suffolk sheep), lane 3: #294 (G3571-inoculated Corriedale sheep), Mo: mouse-adapted scrapie Obihiro (25 µg brain equivalent), b: classical natural BSE (C-BSE) (0.5 mg brain equivalent). A and D. PrPres was detected using mab T2. PrPres in lanes 1, and 2 was classified as high-molecular-weight PrPres (h-type PrPres), and that in lanes 3 was classified as low-molecular-weight PrPres (l-type PrPres). B and E. PrPres was detected using mab SAF-84. A 14-kDa fragment of PrPres (PrPres #2) was detected in #294. C. PrPres was detected using mab P4. PrPres was analyzed before (A, B and C) or after (D and E) PNGase F deglycosylation. Size markers (in kDaltons) are indicated on the left.

**Figure 2:**
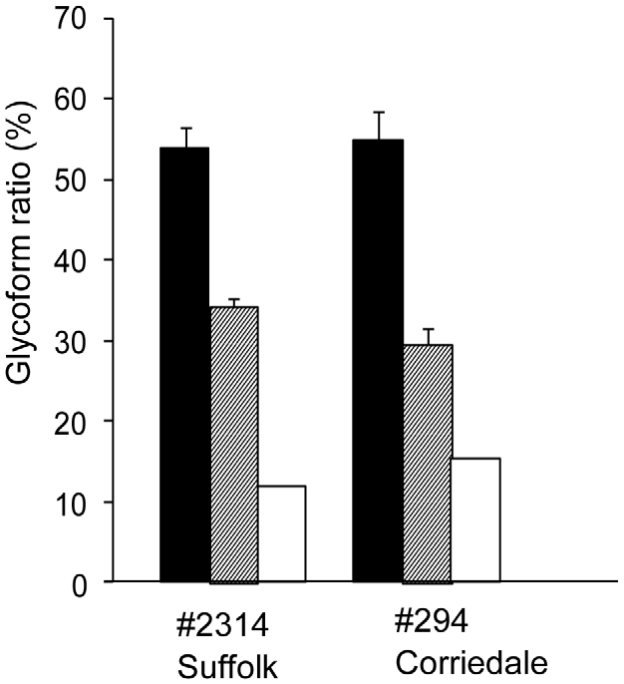
Glycoform profile of PrPres in the sheep brain. Glycoforms of PrPres that accumulated in the experimentally scrapie-challenged sheep were analyzed. We analyzed the signal intensity of the bands shown in Fig. 1A. PrPres was detected by mab T2. Black bar: Ratio of diglycosylated PrPres to total PrPres. Dashed bar: Monoglycosylated PrPres. White bar: Non-glycosylated PrPres. Values are expressed as the mean and standard deviation (percentage).

PrPres accumulation in the brains of sheep experimentally challenged with scrapie was examined by Western blot. Three PrPres bands were detected from the brain tissues of both sheep. Analysis with a mab T2, showed that the molecular weight of unglycosylated PrPres from the Suffolk sheep was similar to that of PrPres from G3571 (Fig. 1A) and scrapie-adapted mice. In contrast, the molecular weight of unglycosylated PrPres from the Corriedale sheep was lower than that of the PrPres from the Suffolk sheep and that of the PrPres causing classical BSE (C-BSE) (Figs. 1A and D). Analysis using mab P4 showed a different immunoreactivity of PrPres from the Corriedale sheep than that of Suffolk sheep (Fig. 1C). However, no distinct differences in the glycoforms of PrPres were detected between the Suffolk and Corriedale sheep by analysis using mab T2 (Fig. 2). We also tested for C-terminally cleaved PrPres fragments by using mab SAF-84. An additional 14-kDa PrPres product was clearly detected from the Corriedale sheep. This 14-kDa band was also detected after PNGase F digestion (Fig. 1E). However, only a small amount of the 14-kDa band was present in the original G3571 sheep and the inoculated Suffolk sheep and in BSE-affected cattle (Figs. 1B and S1).

### Characterization of PrPres in different regions of the sheep brain

**Figure 3:**
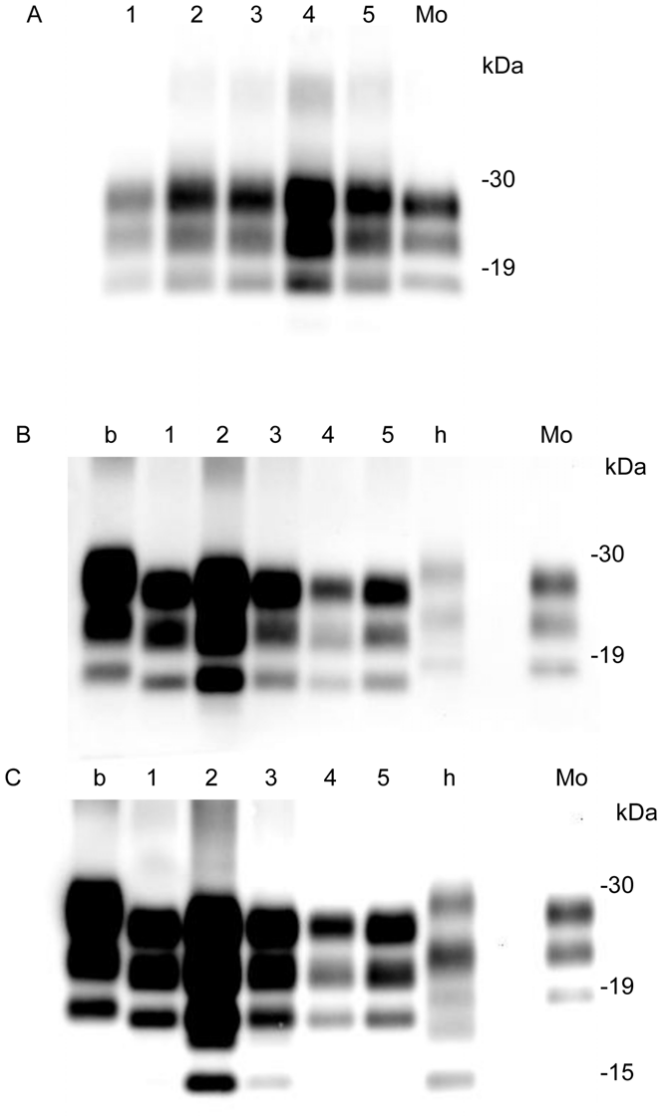
PrPres detection from different regions of the sheep brain. Each lane contained 0.5 mg sheep brain equivalent sample. Lane 1: cerebral cortex, lane 2: brainstem (pons), lane 3: cerebellar medulla, lane 4: cerebellar cortex, lane 5: obex, Mo: mouse-adapted scrapie (25 µg brain equivalent). b: C-BSE (0.5 mg brain equivalent), h: H-type atypical BSE (0.5 mg brain equivalent). PrPres from (A) #2314 (Suffolk sheep) and (B) #294 (Corriedale sheep) was detected using mab T2. C. PrPres from #294 was detected by mab SAF-84.

Both sheep harbored PrPres in the brain areas examined. In the Suffolk sheep, the PrPres signal was high in the cerebellar cortex (Fig. 3A, lane 4); moderate in the brainstem, cerebellar medulla, and obex (Fig. 3A, lanes 2, 3 and 5); and low in the cerebral cortex (Fig. 3A, lane 1). Mab 6H4, which recognizes the subregion 147–155 of sheep PrP showed a similar result with that of mAb T2 (data not shown). In contrast, in the Corriedale sheep, the PrPres concentration was high in the brainstem (pons) (Fig. 3B, lane 2) and low in the cerebellar cortex (Fig. 3B, lane 4). The 14-kDa band signals detected using SAF-84 were strong in the brainstem (Fig. 3C, lane 2), weak in the cerebral medulla, and faint in the cerebral cortex, cerebellar cortex and obex (Figs. 3 and S2).

### Immunohistochemical analysis of PrP^Sc^ in the sheep brain

**Figure 4:**
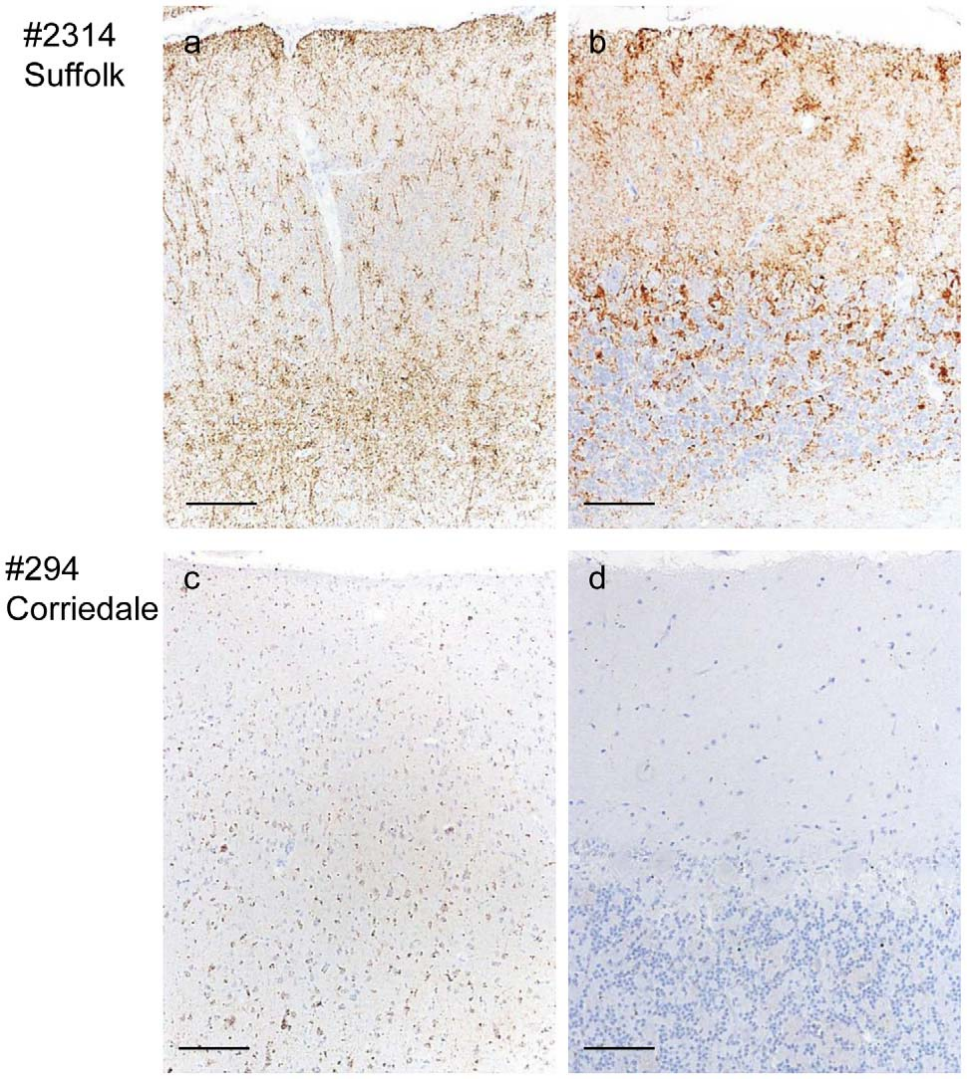
Analysis by neuropathology of experimentally scrapie-challenged sheep. Immunohistochemical analysis: a. frontal cortex, #2314; b. cerebellum, #2314; c. frontal cortex, #294; and d. cerebellum, #294. PrP^Sc^ immunolabeling was performed using mab T1. Bar: 100 µm.

In general, vacuolation and PrP^Sc^ accumulation in all brain areas was more severe in the Suffolk sheep than in the Corriedale sheep (Figs. 4 and S3). In the former, extracellular stellate, perineuronal, and punctuate PrP^Sc^ deposits were seen in the cerebral and cerebellar cortices, thalamus, and brainstem (Fig. 4). In contrast, fine intracellular PrP^Sc^ deposits, particularly within the neurons and glial cells, and near-total absence of extracellular PrP^Sc^ deposits were observed in the Corriedale sheep (Fig. 4).

### PrPres accumulation in the peripheral tissues of sheep

PrPres was detected in the peripheral nerve tissues, lymphoid tissues, and adrenal gland of the Suffolk sheep (Table 2 and Fig. S4). In contrast, in the Corriedale sheep, PrPres accumulation in peripheral nerve tissues was limited to the optic and vagus nerves (weakly positive). A large amount of PrPres had accumulated in the lymphoid tissues, and the molecular weight differed from that of the PrPres in the nervous tissues. No PrPres was detected in the adrenal gland (Table 2 and Fig. S4).

**Table 2:** Summary of PrPres distribution in scrapie-challenged sheep.

Western blot lane		Tissue	Sheep	
			Suffolk	Corriedale
			#2314	#294
Fig. 3	1	Cerebral cortex	+	++
	2	Brainstem (pons)	++	+++
	3	Cerebellar medulla	++	++
	4	Cerebellum cortex	+++	+
	5	Obex	++	+++
Fig. S4	1	Trigeminal ganglia	+++	-
	2	Stellate ganglia	-	-
	3	Vagosympathetic trunk	+	-
	4-5	Vagus nerve	+	±
	6	Accessory nerve	+++	-
	7	Brachial nerve plexus	-	-
	8	Median nerve	+	-
	9	Radial nerve	+	ND
	10	Phrenic nerve	±	ND
	11	Sciatic nerve	+	-
	12	Optic nerve	+++	++
	13	Retina	+++	++
	14	Pituitary gland	++	+
	15	Spleen	++	+
	16	Tonsil	+++	+++
	17	Retropharyngeal lymph node	+	+++
	18	Mandibular lymph node	+	+++
	19	Anterior mediastinal lymph node	±	++
	20	Anterior cervical lymph node	+++	++
	21	Subiliac lymph node	±	++
	22	Popliteal lymph node	+	++
	23	Hepatic lymph node	+++	-
	24	Internal iliac lymph node	+++	±
	25	External iliac lymph node	-	-
	26	Mesenteric lymph node	±	-
	27	Renal lymph node	±	++
	28	Thymus	±	-
	29	Spinal cord	+++	++
	30	Parotid gland	-	-
	31	Mandibular gland	-	-
	32	Thyroid gland	-	-
	33	Liver	-	-
	34	Kidney	-	-
	35	Pancreas	-	-
	36	Adrenal gland	+	-

+++, ++, +, ±, - indicate the signal intensity on Western blot.

ND: not done.

### Comparison of the PrPres observed in the nervous and lymphoid tissues of the Corriedale sheep

**Figure 5:**
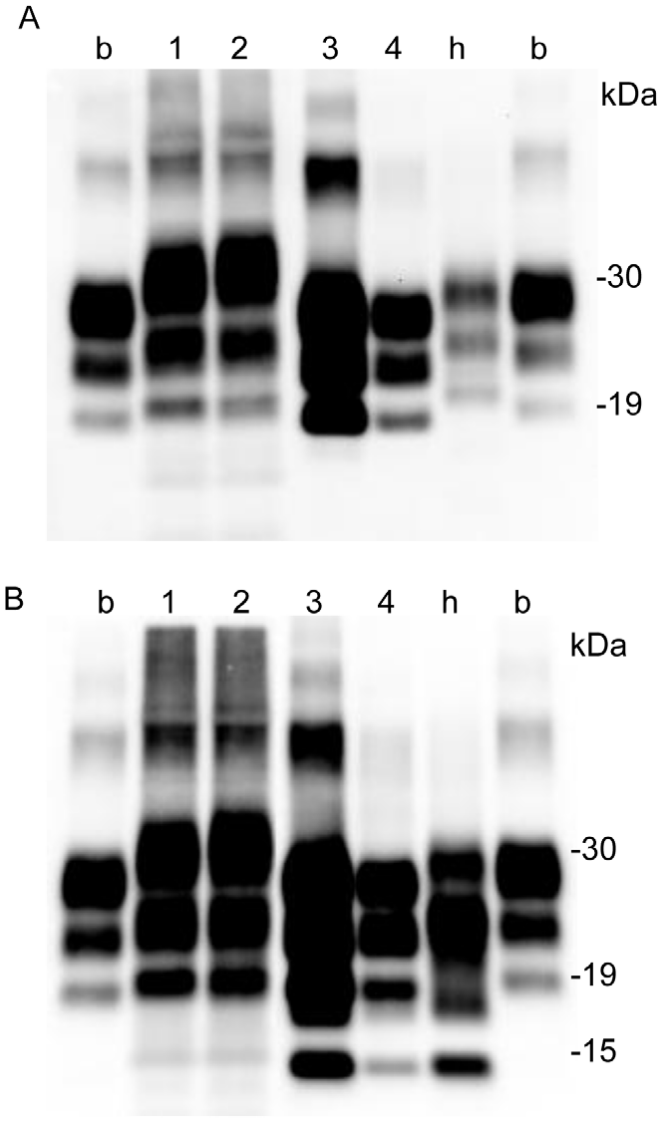
PrPres profile in the nervous and lymphoid tissues of #294 (Corriedale sheep). Lane 1: anterior cervical lymph node, lane 2: mesenteric lymph node, 3: optic nerve, and 4: brainstem. b: C-BSE, h: H-type atypical BSE. A. PrPres was detected using mab T2. In #294, l-type PrPres was detected from the optic nerve and brainstem, whereas h-type PrPres was detected from the lymphoid tissues. B. PrPres was also detected using mab SAF-84. An additional 14-kDa band was detected from l-type PrPres, but not from h-type PrPres.

The molecular weight of unglycosylated PrPres in the lymphoid tissues of the Corriedale sheep was greater than that of C-BSE PrPres (Fig. 5A, lanes 1 and 2). In contrast, the molecular weight of the PrPres in the nervous tissues was slightly lower than that of C-BSE PrPres (Fig. 5A, lanes 3 and 4). The 14-kDa fragment was detected from the optic nerve and brainstem, but not from the lymphoid tissues (Fig. 5B).

### Interspecies transmission of scrapie to mice

G3571-inoculated ICR mice in the primary, secondary and third passages showed incubation periods of 412, 315.8 and 337 d, respectively (Table 3). Wild-type mice inoculated with the brain homogenate obtained from the Suffolk sheep showed clinical signs at 413.5 d after inoculation; this period is similar to the incubation period for G3571. In contrast, wild-type mice inoculated with the brain homogenate obtained from the Corriedale sheep showed no clinical signs even at 700 d after inoculation, and no PrPres accumulation was observed in the brains of these mice. Then the brain homogenate from both sheep were intracerebrally inoculated into bovinized transgenic mice (TgBoPrP). After the first passage, TgBoPrP mice inoculated with G3571-brain showed clinical signs after 249.5 d, and in the second passage, showed signs after 184.6 d. A similar incubation period was observed in mice inoculated with samples from the Corriedale sheep. However, in the case of the Suffolk sheep, the incubation period for the TgBoPrP mice was 693.8 d in the primary passage and 457.7 d in the second passage. The numbers of mice used for each inoculation is shown in Table 3.

**Table 3:** Incubation period in mice inoculated with sheep scrapie.

Inoculum	Passage^1^	Wild-type mice				TgBoPrP			
		n/n_0_ 2	Incubation period	PrPres profle^3^	PrPres# 2^4^	n/n_0_	Incubation period	PrPres profle	PrPres #2
G3571(Suffolk)	1st	6/6	412.0±24.9^5^	h-type PrPres	-	6/6	249.5±45.8	l-type PrPres	-
	2nd	5/5	315.8±10.0	h-type PrPres	-	7/7	184.6±4.4	l-type PrPres	-
	3rd	5/5	337.0±30.1	h-type PrPres	-		Not done		
#2314(Suffolk)	1st	6/6	413.5±31.0	h-type PrPres	-	6/6	693.8±62.8	l-type PrPres	-
	2nd		Not done			7/7	457.7±9.1	l-type PrPres	-
#294(Corriedale)	1st	0/6	>700	No PrPres		5/6	250.6±3.3	l-type PrPres	-
	2nd		Not done			7/7	181.4±9.1	l-type PrPres	-

Brain homogenates of sheep with scrapie (G3571, #2314, and #294) were intracerebrally inoculated into wild-type (ICR) mice and TgBoPrP mice.

1 Brain homogenates from diseased mice were inoculated into other mice.

2 Diseased mice/inoculated mice.

3 Classified on the basis of the molecular weight of unglycosylated PrPres.

4 Existence of 14-kDa of PrPres fragment.

5 Mean incubation period ± standard deviation (days).

The findings for neuropathology in TgBoPrP mice after inoculation with samples from the G3571 and the Corriedale sheep were similar. Inoculation of these mice with samples from the Suffolk sheep produced a different lesion profile (Fig. S5). However, diffuse PrP^Sc^ deposition was observed in all TgBoPrP mice, and no significant differences were observed in the findings of immunohistochemical analysis (data not shown).

### PrPres analysis in scrapie-affected mice

**Figure 6:**
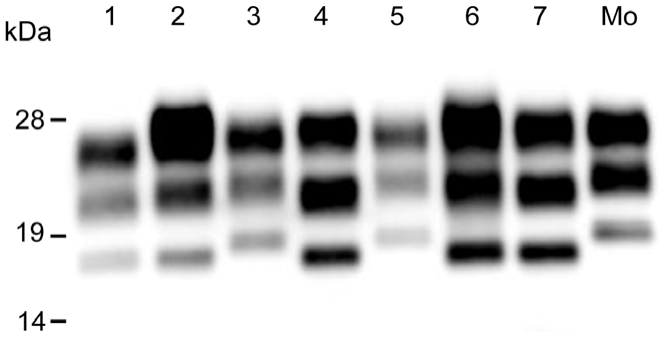
PrPres analysis in scrapie-passaged mice. PrPres that accumulated in the brains of scrapie-passaged mice was analyzed by Western blot. Lanes 1 and 2: C-BSE-passaged mice, lanes 3 and 4: G3571-passaged mice, lanes 5 and 6: mice passaged with samples from #2314 (Suffolk), lane 7: mice passaged with samples from #294 (Corriedale). Lanes 1, 3, 5: ICR mice. Lanes 2, 4, 6 and 7: TgBoPrP mice. Mo: mouse-adapted scrapie. PrPres was detected using mab SAF-84.

The molecular weight of the PrPres that accumulated in the ICR mice infected with samples from the G3571 and Suffolk sheep was greater than that of C-BSE PrPres (Fig. 6, lanes 3 and 5). However, the molecular weight of the PrPres in the TgBoPrP mice challenged with the 3 scrapie strains was lower than that of mouse scrapie strains, but similar to that of C-BSE PrPres (Fig. 6, lanes 4, 6 and 7). The 14-kDa PrPres band was not detected from TgBoPrP mice infected with samples from the Corriedale sheep (Fig. 6, lane 7).

## Discussion

Prion pathology varies with the host species. PrP amino acid polymorphisms are attributed to the susceptibility of scrapie and their incubation periods [27], [28], [29]. Multiple prion strains have been isolated from some cases of natural sheep scrapie [30], [31]. In this study, we detected 2 different scrapie phenotypes in different sheep breeds, even though they had identical PrP genotypes. One of these strains, the unusual CH1641 isolate, has been isolated from both natural and experimental cases of sheep scrapie [15], [32], [33]. This strain shows some molecular similarities to BSE-causing strains, and further studies are required to clarify its possible involvement in the development of BSE and atypical BSE. In the case of CH1641 prions, both l-type PrPres and the 14-kDa PrPres #2 are detected, and PrP^Sc^ are mainly accumulated within the neurons of affected sheep, and very little extracellular PrP^Sc^ accumulation is seen in immunohistochemistry [34]. These characteristics were identical to the Corriedale sheep (Fig. 4), and we have concluded that the Corriedale sheep prions were CH1641-like prions. In the Corriedale sheep, l-type PrPres with and without PrPres #2 was observed in the brain (Fig. 3C). A small amount of PrPres #2 band was detected in the cerebellum of the Corriedale sheep (Fig. S2). In this study, prominent 14-kDa band was detected in the Corriedale, but only a small amount in the Suffolk sheep (Fig. 1). This result is consistent with the previous report, that the l-type PrP^Sc^ could be present in a number of scrapie sources [18]. Interestingly, the faint PrPres #2 band was also detected from BSE cattle (Fig. S1). Multiple PrP^Sc^ types have been detected in the brains of CJD patients [35], [36]. It has been shown that cattle intracerebrally inoculated with scrapie showed the two disease phenotypes (h-type PrPres and l-type PrPres). In some of the inoculated cattle, PrPres profile was different based on what brain region was obtained [37].

CH1641-like PrPres (l-type PrPres) was not detected in the lymphoid tissues of the Corriedale sheep by Western blot. However, the prions accumulated in lymphoid tissues but were almost completely absent in peripheral nervous tissues (Table 2 and Fig. S4). In contrast, PrPres was detected from both the lymphoid tissues and peripheral nervous tissues of the Suffolk sheep, which exhibited signs of typical scrapie (Table 2 and Fig. S4). Prions are thought to be transmitted from the peripheral tissues to the brain via the peripheral nerves [38], [39]. In this experiment, prions were intravenously inoculated and therefore had easy access to the lymphoid tissues. It has been reported that the scrapie strain in the sheep might influence the resulting PrP phenotype pathology, and PrP^Sc^ accumulation in lymphoid tissues is unrelated to the route of infection [40]. We consider that the inability of PrP^Sc^ to replicate in the peripheral nervous tissues leads to inefficient neuroinvasion and therefore longer incubation periods (Table 1). PrPres in the lymphoid tissues of the Corriedale sheep was classified as the h-type on the basis of the molecular weight of its unglycosylated form and the absence of PrPres #2 (Fig. 5B). A possible explanation is that PrP^Sc^ conversion differs in different tissues, leading to differences in PrP^Sc^ propagation: Corriedale sheep may have different prion strains in the CNS and lymphoid tissues. A similar result was obtained from the spleen of ovinized transgenic mice infected with the CH1641 [41]. We inoculated intravenously 2 other Suffolk sheep. Scrapie was successfully transmitted to 2 sheep (one Suffolk and one Corriedale), and we could not completely exclude that prion strain separation was caused by a matter of chance or resulted from an artificial route of transmission. Additional studies are required to analyze the detailed characteristics of prions accumulated in the peripheral tissues of Corriedale sheep. A transmission study of lymphoid tissues to mice is in progress.

**Figure 7:**
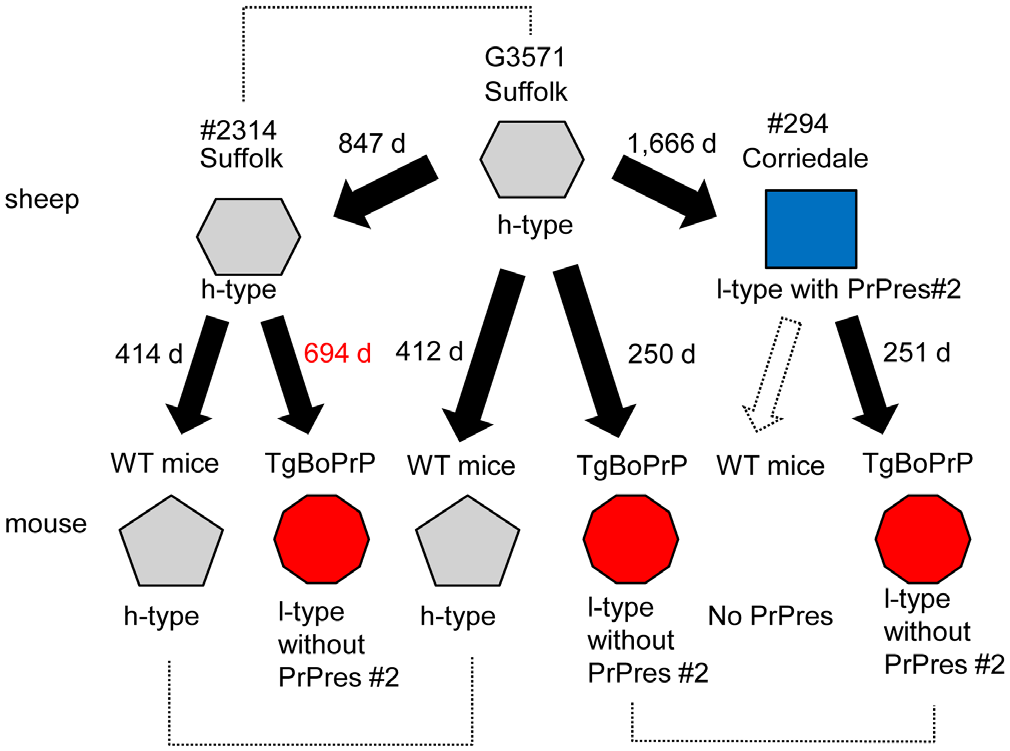
Illustrative models of dominant PrPres in the brains of sheep and mice. PrPres was classified on the basis of its molecular weight (h-type or l-type) and the existence of the PrPres #2 fragment. The dashed lines indicate similar PrPres. In the case of sheep transmission, PrPres in #2314 was similar to that in G3571. In the case of mouse transmission, similar PrPres was amplified in wild-type mice inoculated with samples from #2314 and G3571. All the 3 scrapie-passaged TgBoPrP mice harbored similar PrPres in their brains. Prions from #294 were not transmitted to wild-type mice. The mean incubation period (days) is indicated beside the arrows. WT mice: wild-type mice.

A mouse transmission study showed that the G3571 strain contained different prions (Fig. 7). The h-type PrPres was detected in samples from both G3571 and #2314 Suffolk, when wild-type ICR mice were inoculated. In addition, both showed similar incubation periods (approximately 410 d) and findings by neuropathology for the presence of PrP plaques (data not shown). However, no PrPres was detected in ICR mice inoculated with samples from the Corriedale sheep. These results indicate that h-type, but not l-type, PrPres was transmitted to wild-type ICR mice. Since CH1641 prions cannot be transmitted to wild-type mice [13], [32], we could not compare the PrPres phenotypes of the 3 sheep scrapie strains in wild-type mice. We then used TgBoPrP mice for this analysis and to determine the relationship between BSE strains and the strain infecting the Corriedale sheep. The results of the transmission study in TgBoPrP mice differed from those obtained in the case of wild-type mice. All the scrapie-passaged TgBoPrP mice harbored identical l-type PrPres without PrPres #2, but showed different incubation periods: approximately 250 d in the case of the G3571 and Corriedale strains, and 694 d in the case of the Suffolk strain (Fig. 7 and Table 3). These results differ from those obtained in the case of the sheep and wild-type mice (Fig. 7). The incubation period of G3571-passaged wild-type mice (412 d) was similar to that of wild-type mice passaged with prions from the Suffolk sheep, and that of G3571-passaged TgBoPrP mice (250 d) was identical to that of TgBoPrP mice passaged with prions from the Corriedale sheep. The different incubation period (694 d) in TgBoPrP mice passaged with prions from the Suffolk sheep also indicates that multiple prions coexist in sheep (Fig. 7). Alternatively, there is a possibility that the 2 sheep respond differently to a single scrapie isolate. It has reported that PrPres conformers were selected by prion adaptation in interspecies transmission [42]. There may have been a difference in the proportion of the l-type associated strain that could have been decreased in the #2314 Suffolk compared to that in the G3571 sheep and that the longer incubation period may just reflect a lower infectious titer of the l-type strain. These results indicate that prion propagation is influenced by host species. Further, the transmission study using ovinized PrP expressing mice is under consideration, and it may help to solve this question.

Our results show that sheep scrapie is cause by multiple prion strains, and the resultant phenotype depends on which prions are dominantly propagated in a given host. The reason why multiple prion strains emerged during intra- and interspecies transmission in this study is unclear. Differences between unidentified prion-related host factors in the Corriedale and Suffolk sheep may have influenced the neuropathological findings and PrPres distribution. A putative cofactor, designated protein X, is thought to be required for PrP^Sc^ formation and may be involved in the experiments here [43]. Our results indicate that the host PrP^C^ and other factors may control prion pathogenesis.

It is possible that the efficiency of prion replication in peripheral tissues is low, leading to selection and/or mutations of the non-dominant prions. This phenomenon may be augmented by interspecies transmission and thereby result in the propagation of minor prions in the infected animals. Intracerebral inoculation may decrease the chances of strain selection and efficiently convert PrP^C^ to PrP^Sc^ in the brains of infected animals. The conversion of PrP^C^ to PrP^Sc^ is possibly a multi-step process involving the formation of several intermediate forms of PrP [20], [44]; one such intermediate form of PrP^Sc^ may have been selected in the affected animals.

All scrapie prions propagated in TgBoPrP mice showed different characteristics from those of C-BSE and L-BSE prions [45], [46]. However, the SAF-84-detected PrPres profile of the Corriedale sheep resembled that of H-BSE with the existence of 14 kDa band (Fig. 5). A comparative study of these 2 prions will be a good model for the analysis of C-terminal truncated PrPres fragments and their pathogenesis. The l-type PrPres and PrPres #2 have been detected from ovine transgenic mouse affected with CH1641 prions [15]. We did not detect PrPres #2 in the affected TgBoPrP mice. CH1641-like prions may induce different pathogenetic processes in cattle and in sheep.

The origin of atypical BSEs is unknown. If atypical BSEs result from sporadic BSE [47], feed-ban programs will need to be imposed. It has been proposed that the BSE may have originated from sheep scrapie [48]. Our results clearly show that unusual prions were masked by typical prions. Prion strain selection can occur not only after interspecies transmission but also after intraspecies transmission in animals with identical PrP genotypes. The significance of prion strains as defined in mice remains poorly understood in sheep scrapie. This study may bring some new insights in this question.

## Supporting Information

Figure S1 Western blot analysis of PrPres in scrapie sheep brain. Obex homogenates were subjected to Western blot analysis. Each lane contained 1.0 mg sheep brain equivalent sample. Lane 1: G3571, lane 2: #2314 (G3571-inoculated Suffolk sheep), lane 3: #294 (G3571-inoculated Corriedale sheep), Mo: mouse-adapted scrapie Obihiro (25 µg brain equivalent), b: classical natural BSE (C-BSE). PrPres was detected using mab SAF-84. A faint 14-kDa fragment of PrPres (PrPres #2) was detected in Suffolk by prolonged exposure of the same membrane as that depicted in Fig. 1B. Size markers (in kDaltons) are indicated on the left. (TIF)

Figure S2 **PrPres of #294 (Corriedale sheep).** Lane 1: cerebral cortex, lane 2: brainstem (pons), lane 3: cerebellar medulla, lane 4: cerebellar cortex, lane 5: obex, Mo: mouse-adapted scrapie, b: C-BSE, h: H-type atypical BSE. PrPres was detected using mab. SAF-84. The difference in PrPres#2 distribution was shown by prolonged exposure of the same membrane as that depicted in Fig. 3C. Mab 44B1 [49], that recognizes the subregion 159–234 of sheep PrP showed a similar result to that of SAF-84 (data not shown). (TIF)

Figure S3 PrP^Sc^ distribution in experimental sheep. Immunohistochemical analyses of experimentally challenged sheep: #2314 (Suffolk) and #294 (Corriedale). Left: thalamus and hypothalamus, right: cerebellum. PrP^Sc^ immunolabeling was achieved using mabT1. (TIF)

Figure S4 PrPres distribution in the peripheral tissues of #2314 (Suffolk sheep) (A) and #294 (Corriedale sheep) (B). Lane 1: trigeminal ganglia, 2: stellate ganglia, 3: vagosympathetic trunk, 4 and 5: vagus nerve, 6: accessory nerve, 7: brachial nerve plexus, 8: median nerve, 9: radial nerve, 10: phrenic nerve, 11: sciatic nerve, 12: optic nerve, 13: retina, 14: pituitary gland, 15: spleen, 16: tonsil, 17: retropharyngeal lymph node, 18: mandibular lymph node, 19: anterior mediastinal lymph node, 20: anterior cervical lymph node, 21: subiliac lymph node, 22: popliteal lymph node, 23: hepatic lymph node, 24: internal iliac lymph node, 25: external iliac lymph node, 26: mesenteric lymph node, 27: renal lymph node, 28: thymus, 29: spinal cord, 30: parotid gland, 31: mandibular gland, 32: thyroid gland, 33: liver, 34: kidney, 35: pancreas, 36: adrenal gland, Mo: mouse-adapted scrapie. Note that in sheep #2314 (Suffolk), most of the peripheral nervous and lymphoid tissues harbored PrPres. In sheep #294 (Corriedale), the spinal cord, vagus nerve, optic nerve, retina, spleen, and several lymph nodes were positive for PrPres. (TIF)

Figure S5 Lesion profile of scrapie-passaged TgBoPrP mice. Vacuolation in each brain region was scored on a scale of 0–5 (mean values). 1, dorsal medulla; 2, cerebellar cortex; 3, superior cortex; 4, hypothalamus; 5, thalamus; 6, hippocampus; 7, septal nuclei of the paraterminal body; 8, cerebral cortex at the levels of the hypothalamus and thalamus; and 9, cerebral cortex at the level of the septal nuclei of the paraterminal body [50]. Filled circles: G3571-affected TgBoPrP mice, filled squares: #2314-affected TgBoPrP mice, open circles: #294-affected TgBoPrP mice. The numbers of mice used for each analysis is shown in Table 3 (n = 6 or 7). (TIF)
