# Supplementary figures and images for: Intraspecies Prion Transmission Results in Selection of Sheep Scrapie Strains
Source: PLoS One. 2010 Nov 16;5(11):e15450. doi: 10.1371/journal.pone.0015450 (PMC2982847; doi:10.1371/journal.pone.0015450)

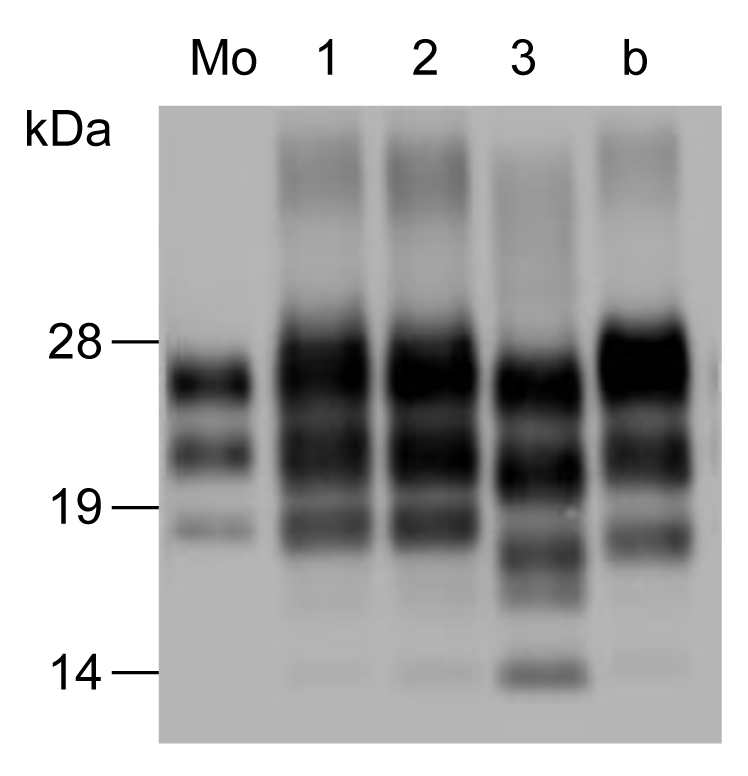

Supplement: Figure S1 — Western blot analysis of PrPres in scrapie sheep brain. Obex homogenates were subjected to Western blot analysis. Each lane contained 1.0 mg sheep brain equivalent sample. Lane 1: G3571, lane 2: #2314 (G3571-inoculated Suffolk sheep), lane 3: #294 (G3571-inoculated Corriedale sheep), Mo: mouse-adapted scrapie Obihiro (25 µg brain equivalent), b: classical natural BSE (C-BSE). PrPres was detected using mab SAF-84. A faint 14-kDa fragment of PrPres (PrPres #2) was detected in Suffolk by prolonged exposure of the same membrane as that depicted in Fig. 1B. Size markers (in kDaltons) are indicated on the left. (TIF) [file pone.0015450.s001.tif]

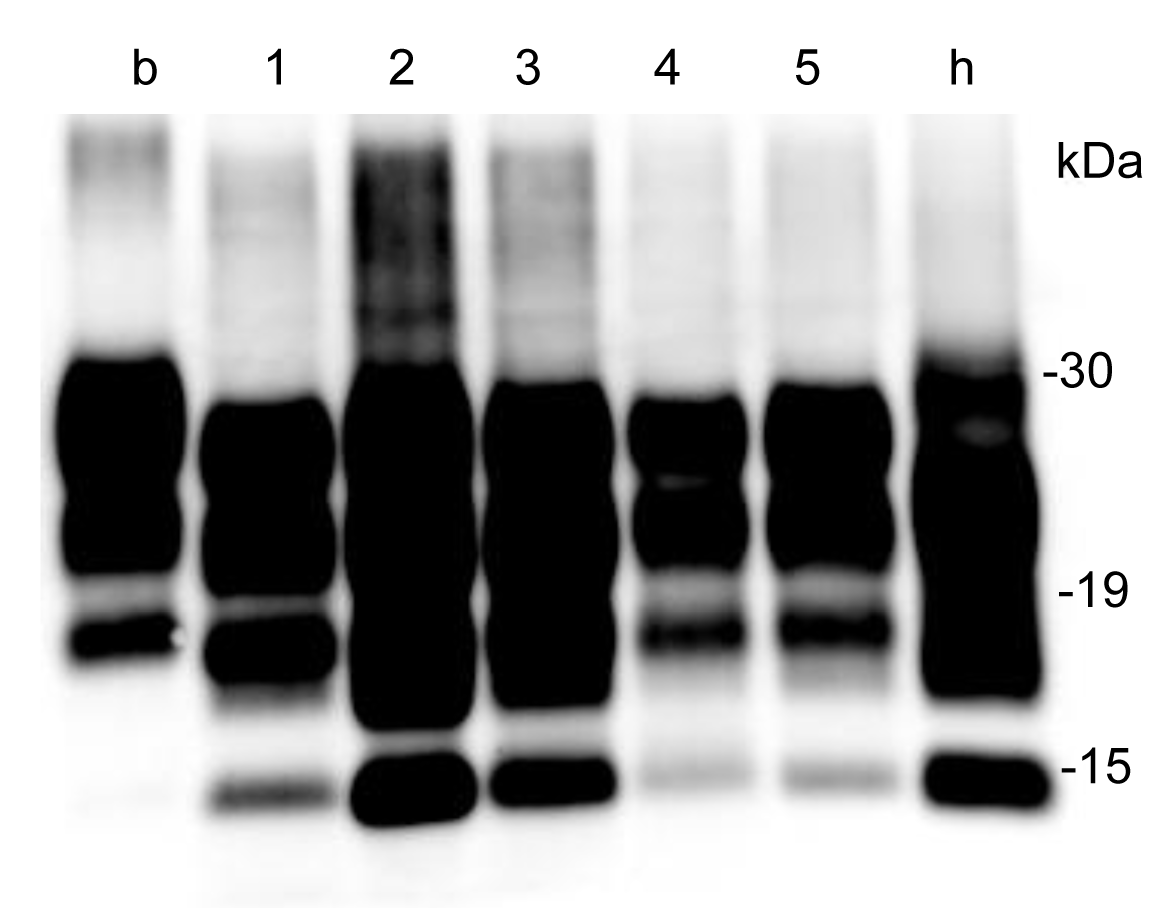

Supplement: Figure S2 — PrPres of #294 (Corriedale sheep). Lane 1: cerebral cortex, lane 2: brainstem (pons), lane 3: cerebellar medulla, lane 4: cerebellar cortex, lane 5: obex, Mo: mouse-adapted scrapie, b: C-BSE, h: H-type atypical BSE. PrPres was detected using mab. SAF-84. The difference in PrPres#2 distribution was shown by prolonged exposure of the same membrane as that depicted in Fig. 3C. Mab 44B1 [49], that recognizes the subregion 159–234 of sheep PrP showed a similar result to that of SAF-84 (data not shown). (TIF) [file pone.0015450.s002.tif]

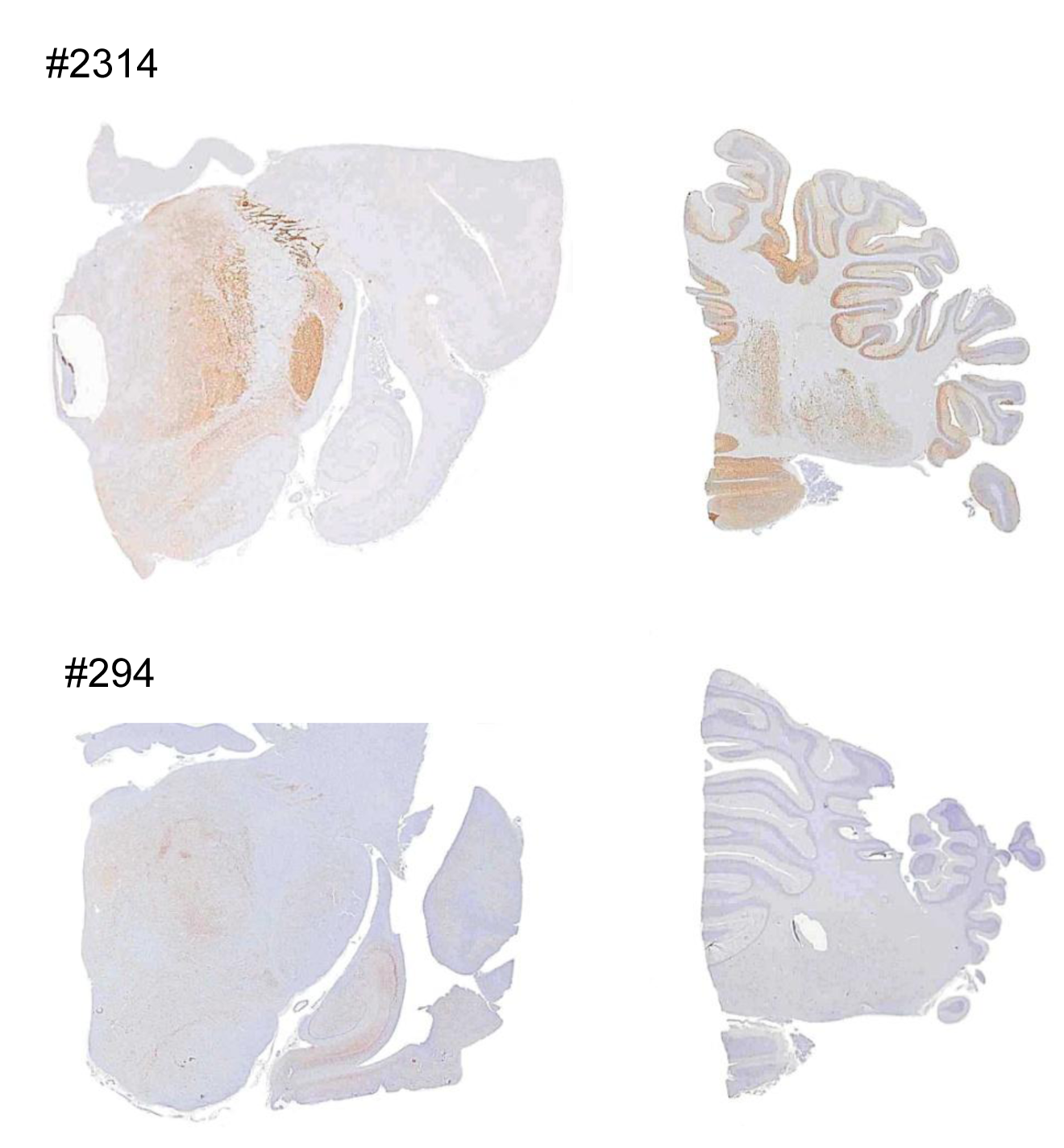

Supplement: Figure S3 — PrPSc distribution in experimental sheep. Immunohistochemical analyses of experimentally challenged sheep: #2314 (Suffolk) and #294 (Corriedale). Left: thalamus and hypothalamus, right: cerebellum. PrPSc immunolabeling was achieved using mabT1. (TIF) [file pone.0015450.s003.tif]

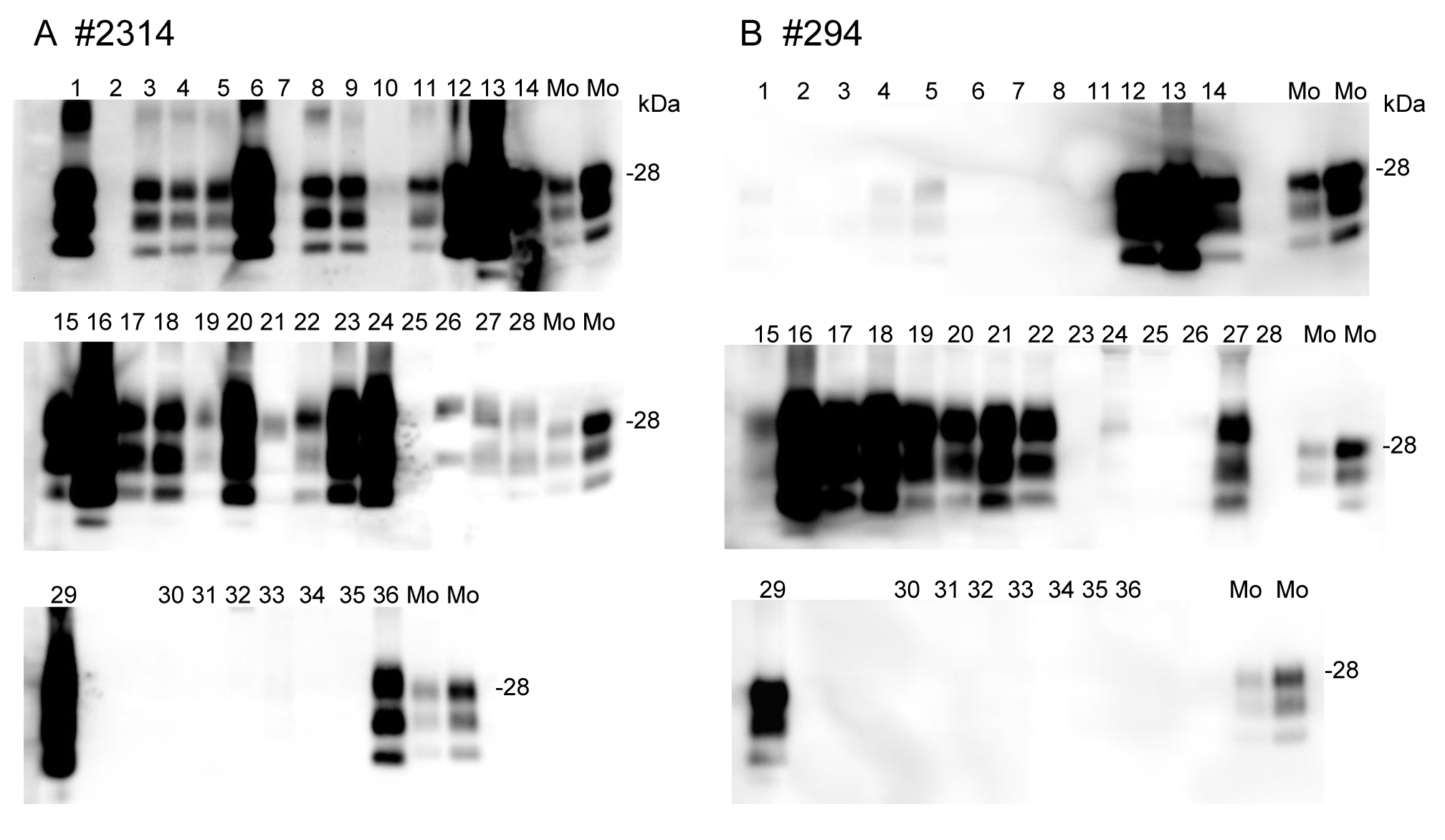

Supplement: Figure S4 — PrPres distribution in the peripheral tissues of #2314 (Suffolk sheep) (A) and #294 (Corriedale sheep) (B). Lane 1: trigeminal ganglia, 2: stellate ganglia, 3: vagosympathetic trunk, 4 and 5: vagus nerve, 6: accessory nerve, 7: brachial nerve plexus, 8: median nerve, 9: radial nerve, 10: phrenic nerve, 11: sciatic nerve, 12: optic nerve, 13: retina, 14: pituitary gland, 15: spleen, 16: tonsil, 17: retropharyngeal lymph node, 18: mandibular lymph node, 19: anterior mediastinal lymph node, 20: anterior cervical lymph node, 21: subiliac lymph node, 22: popliteal lymph node, 23: hepatic lymph node, 24: internal iliac lymph node, 25: external iliac lymph node, 26: mesenteric lymph node, 27: renal lymph node, 28: thymus, 29: spinal cord, 30: parotid gland, 31: mandibular gland, 32: thyroid gland, 33: liver, 34: kidney, 35: pancreas, 36: adrenal gland, Mo: mouse-adapted scrapie. Note that in sheep #2314 (Suffolk), most of the peripheral nervous and lymphoid tissues harbored PrPres. In sheep #294 (Corriedale), the spinal cord, vagus nerve, optic nerve, retina, spleen, and several lymph nodes were positive for PrPres. (TIF) [file pone.0015450.s004.tif]

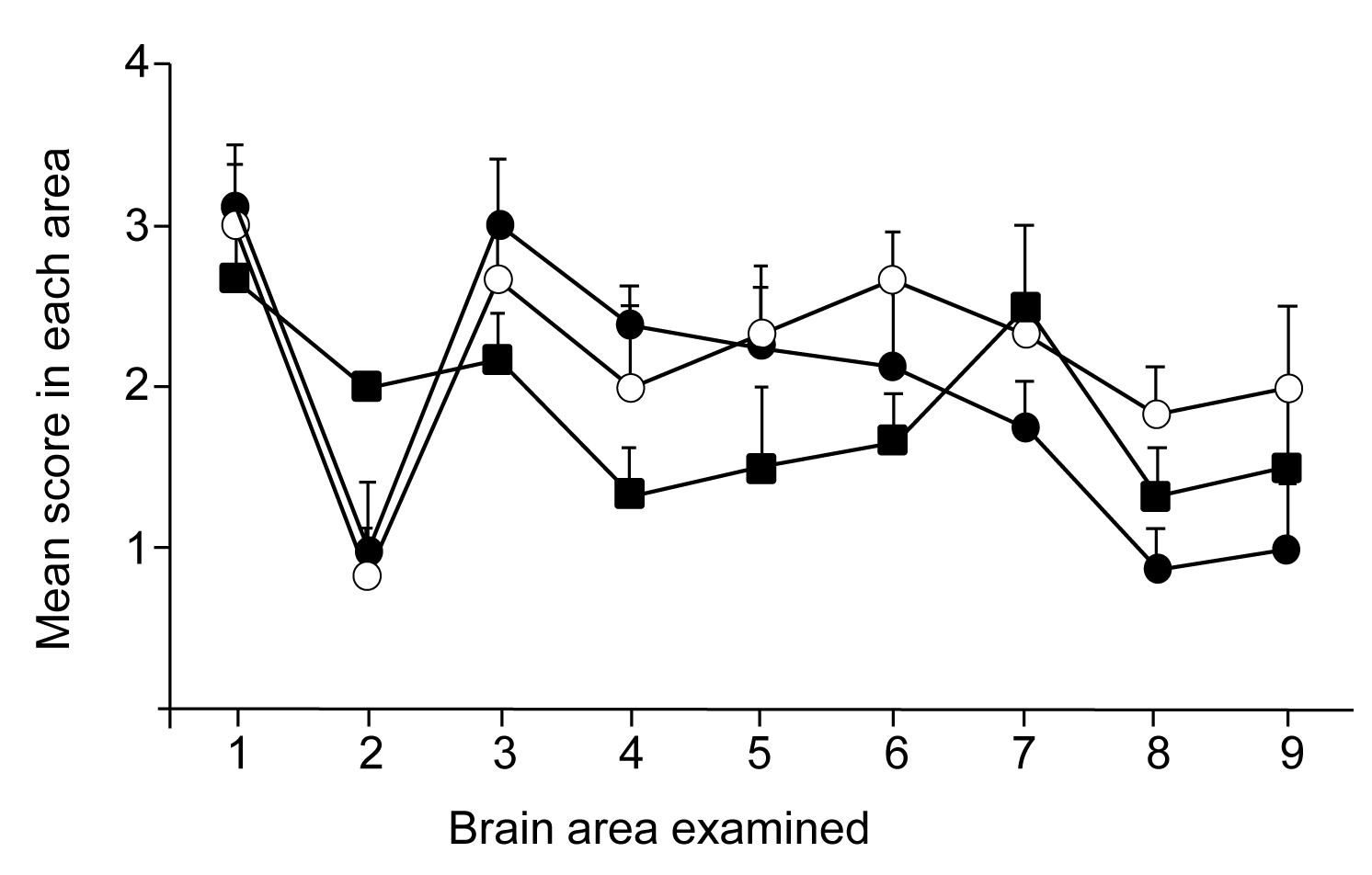

Supplement: Figure S5 — Lesion profile of scrapie-passaged TgBoPrP mice. Vacuolation in each brain region was scored on a scale of 0–5 (mean values). 1, dorsal medulla; 2, cerebellar cortex; 3, superior cortex; 4, hypothalamus; 5, thalamus; 6, hippocampus; 7, septal nuclei of the paraterminal body; 8, cerebral cortex at the levels of the hypothalamus and thalamus; and 9, cerebral cortex at the level of the septal nuclei of the paraterminal body [50]. Filled circles: G3571-affected TgBoPrP mice, filled squares: #2314-affected TgBoPrP mice, open circles: #294-affected TgBoPrP mice. The numbers of mice used for each analysis is shown in Table 3 (n = 6 or 7). (TIF) [file pone.0015450.s005.tif]
